# The Role of Serology Testing in the Context of Immunization Policies for COVID-19 in Latin American Countries

**DOI:** 10.3390/v13122391

**Published:** 2021-11-29

**Authors:** Carlos E. dos Santos Ferreira, Hector Gómez-Dantés, Nancy C. Junqueira Bellei, Eduardo López, Katya A. Nogales Crespo, Miguel O’Ryan, Julieta Villegas

**Affiliations:** 1 Clinical Pathology, Hospital Israelita Albert Einstein, São Paulo 05652-900, Brazil; carlos.ferreira@einstein.br; 2 Microbiology Sector, Federal University of São Paulo’s Central Laboratory Activities, São Paulo 04088-002, Brazil; 3 Brazilian Society of Clinical Pathology and Laboratory Medicine, Rio de Janeiro 22220-040, Brazil; 4 National Institute of Public Health, Cuernavaca 62100, Mexico; hector.gomez@insp.mx; 5 Infectious Diseases Division, Federal University of Sao Paulo, São Paulo 04039-032, Brazil; nbellei@uol.com.br; 6 Department of Medicine, Hospital de Niños Gutiérrez, Buenos Aires C1425-EFD, Argentina; elopez2676@gmail.com; 7 Pediatric Infectious Diseases Program, Faculty of Medicine, University of Buenos Aires, Buenos Aires C1121-ABG, Argentina; 8 Pediatrics and Vaccinology, Faculty of Medicine, University of Salvador, Buenos Aires C1055-AAG, Argentina; 9 Policy Wisdom LLC, Miami, FL 33173, USA; jvillegas@policywisdom.com; 10 Institute of Biomedical Sciences, Faculty of Medicine, University of Chile, Santiago de Chile 8380000, Chile; moryan@med.uchile.cl; 11 Millennium Institute of Immunology and Immunotherapy, University of Chile, Santiago de Chile 8331150, Chile; 12 Chilean Academy of Medicine, Santiago de Chile 6500445, Chile

**Keywords:** COVID-19, SARS-CoV-2, pandemic, serology tests, antibody tests, diagnostic tests, health policy, immunization, vaccination, Latin America

## Abstract

This review aims to explore the role and value of serology testing in the context of COVID-19 immunization policies in Latin American countries and the barriers and challenges to the adequate use and uptake of this tool. It builds on a review of the academic literature, evidence, and existing policies, and includes a multistage process of discussion and feedback by a group of five experts. Regional and country-level evidence and resources from five focus countries—Argentina, Brazil, Chile, Colombia, and Mexico—were collected and analyzed. This review contains an overview of (1) the impact of the SARS-CoV-2 pandemic, the variants of concern and current testing strategies, (2) the introduction of COVID-19 vaccination, (3) the potential use of serology testing to support immunization initiatives, (4) the current frameworks for the use of serology testing in the region, and (5) the barriers and challenges to implementing serology testing in the context of COVID-19 immunization policies, including a discussion on the potential actions required to address these barriers and facilitate the uptake of this strategy in the region. Stakeholders can use elements of this document to guide timely decision-making, raise awareness, and inspire further studies.

## 1. Methodology

Methodologically, this paper builds on a review of literature and policies and a multistage process of discussion, validation, and feedback with a group of five experts from Latin America (LATAM). These experts were selected based on academic merit in various areas of knowledge such as microbiology, epidemiology, public health, pharmacology, and infectiology. An in-depth understanding of serology testing, immunization policies, and the current COVID-19 pandemic was deemed essential. Experience in seroprevalence, seroepidemiology, and post-marketing vaccine effectiveness studies was considered an asset.

This review was inspired by a similar project conducted in Europe [1], adopting a similar framework for the collection of data. As the first step, six primary topics were identified: (1) the impact of the pandemic in the LATAM region and focus countries, (2) current testing strategies, (3) national COVID-19 vaccination policies of focus countries, (4) current policies on the use of serology testing, (5) literature on the use of serology testing in immunization programs, and (6) barriers and challenges limiting implementation.

Evidence on the epidemiological impact was retrieved from well-known monitoring databases, paying particular attention to the countries of interest. Evidence on the socioeconomic impact of the pandemic and the policy frameworks and recommendations for the use of serology testing were extracted from reports of leading international organizations, such as the Organization for Economic Co-Operation and Development, Inter-American Development Bank, International Monetary Fund, Americas Society, Economic Commission for Latin America and the Caribbean, World Health Organization (WHO), Pan-American Health Organization (PAHO), United States Food and Drug Administration (FDA), and Centers for Disease Control and Prevention (CDC). Evidence on the characteristics of serology tests and the use of serology testing in various immunization programs were retrieved from academic publications in peer-reviewed journals (where possible). The national COVID-19 vaccination policies and policies for the use of serology testing of focus countries were retrieved from the official government portals. Finally, the barriers and challenges were identified using all the reviewed sources.

Regional and country-level evidence, policies, and guidelines from five focus countries—Argentina, Brazil, Chile, Colombia, and Mexico—were collected and analyzed. Resources were prioritized using the following inclusion criteria:

- Scientific perspectives on SARS-CoV-2 testing strategies, including challenges and opportunities;
- The health and socioeconomic impact of the pandemic in the region;
- The current position, guidelines, and recommendations on the use of serology testing from key international organizations and focus countries;
- Progress on COVID-19 immunization roll-out and coverage in countries of focus; and
- The national COVID-19 immunization plans and/or strategies of the focus countries.

The information gathered was synthetized and organized, creating a working document. This document served as the basis for the discussion, validation, and feedback provided by all experts during three online panel sessions and rounds of offline review. The working document was edited until the experts reached a consensus.

## 2. Introduction: The Impact of the Pandemic in the Region

On 31 December 2019, the Chinese authorities reported a novel coronavirus causing a cluster of pneumonia-like cases in Wuhan in Hubei Province of China. The virus was later named SARS-CoV-2, and the disease caused by this new virus was named COVID-19 [2]. Between 26 February and 6 March 2020, health officials confirmed cases of COVID-19 in LATAM countries, including Argentina, Brazil, Chile, Colombia, and Mexico [3]. As the new infection spread rapidly across the globe, WHO characterized the COVID-19 outbreak as a pandemic of global proportions on 11 March 2020 [2].

As of 28 October 2021, WHO has reported 244,897,177 confirmed cases of COVID-19, leading to 4,970,429 deaths worldwide [4]. While Europe was profiled as the worst-hit region during the first months of the pandemic, this spot was rapidly overtaken by the Americas. With 93,244,907 confirmed cases and 2,285,843 deaths [4], the Americas is currently profiled as the region with the highest cumulative mortality from COVID-19, accounting for 46% of the total number of deaths worldwide [5]. Within the Americas, LATAM countries have been the worst hit by the pandemic. Whereas reported deaths number about 65 per 100,000 people globally, this increases to 239 deaths per 100,000 people in LATAM [6].

Based on cumulative deaths as of 28 October 2021, the worst affected countries in the region are Brazil (606,679), Mexico (286,888), Peru (200,149), and Colombia (127,159) [6]. Examining the focus countries, Brazil, Colombia, and Argentina have recorded a considerable higher mortality rate compared to Chile and Mexico (see column two Table 1) [6]. However, according to the case fatality ratio, Mexico performs substantially poorer than other countries in the region, recording a 7.6% death ratio for every 100 confirmed COVID-19 cases (which positions the country among the first three globally) [7]. Furthermore, a study in Mexico revealed an excess mortality of 43.1% in 2020 (493,503 deaths attributed to all causes), of which 71.2% are attributed to COVID-19 [8,9].

Nevertheless, it is important to emphasize that as challenges related to the detection and reporting of cases and deaths across the region arise, many have expressed concerns regarding the accuracy of these numbers to reflect the real impact of the pandemic, which considerably limits efforts to compare impact across countries. These challenges include differences in how cases are defined and considerable delays in reporting cases and deaths.

Besides the direct human impact of COVID-19, the pandemic has also severely affected health systems and population health. LATAM countries have experienced severe shortages of essential medicines and equipment to treat COVID-19, personal protective equipment, and health care personnel throughout the pandemic [11,12]. Moreover, due to reduced access to health care, the pandemic has also worsened conditions for those living with chronic illnesses, especially non-communicable diseases [13,14]. Reports have also identified an increase in mental health disorders, particularly anxiety, depression, and suicide [15,16]. In LATAM, these conditions have been found to most commonly affect health care workers and young people [17,18]. The dramatic shift in health priorities has also led to a relocation of health budgets and priorities and a lag in vaccination schedules that target other vaccine-preventable diseases [19,20].

The pandemic has also created socio-political externalities. In many cases, pre-existing social unrest has challenged governments’ capacity to respond to the pandemic comprehensively [21,22]. Concerns about leaders taking advantage to advance their agendas and restrict freedom of expression have also been heard across the region [23].

Measures to combat the pandemic have affected the most vulnerable sectors of society to a larger extent. Reports estimate that approximately 40% of formal workers and 65% of informal workers have no social safety net, including health care coverage [11,24]. In many countries, informal workers, representing up to 60% of the labor market, have struggled to comply with public health measures such as social distancing, quarantines, and mandatory stay-at-home orders [11,24]. Likewise, following hygiene and social distancing measures has proven very difficult for the 21% of Latin America’s urban population that resides in slums, informal settlements, or substandard housing [25,26].

Gender inequalities have also soared during the pandemic. Women, representing 70% of health workers in the region, have been disproportionally exposed to the virus [27]. This has been accompanied by an increased care-related pressure at home [11], deterioration of work–life balance [28], and increased gender-based and domestic violence [28,29]. Finally, school closures have impacted the quality of education and raised concerns with regard to subsequent inequalities on food security, school dropout, and access to the necessary equipment to continue with distance learning in the most vulnerable households [30,31,32].

### Measures to Respond to the SARS-CoV-2 Pandemic in the Region

During the first year of the pandemic, most countries in the region responded with severe containment measures, such as declaring states of emergency and instituting national quarantines between March and October 2020. Evidence suggests that federal states, such as Mexico and Brazil, experienced a delay in implementing such restrictions due in part to the decentralized nature of their governments. Similarly, a closer look at non-pharmaceutical interventions enforced in the countries of focus reveals that, compared to centralized states, federal states enforced fewer restrictions through the central government. In these scenarios, most policies were eventually adopted and adapted by subnational governments [11].

Nevertheless, various non-pharmaceutical interventions, varying substantially in nature and stringency, have been implemented across countries. These interventions include containment and closure policies to reduce the spread of the virus, health system policies to reinforce capacity to manage the pandemic, immunization policies, and economic policies to support vulnerable households and affected sectors of the economy [33].

While all LATAM countries applied containment and closure measures to reduce the spread of the virus, few governments implemented economic policies to protect individuals and businesses and furthermore support adherence to these restrictions. Countries such as Mexico and Paraguay are currently enforcing looser restrictions than Argentina, Chile, Colombia, and Peru [34]. However, health officials recorded a resurgence of COVID-19 cases between April and May 2021, primarily affecting younger population segments. As younger patients are more likely to survive and remain hospitalized for extended periods, there is concern regarding the capacity of health systems to cope with an increase in hospitalizations. Following this trend, PAHO has urged countries in the region to increase the capacity of intensive care units [5].

Implementing non-pharmaceutical interventions that limit mobility and the regular functioning of businesses has sparked fear of an economic crisis. Decisions regarding how to reduce the effects of the pandemic on mortality without compromising the economy any further will continue to be challenging. To date, evidence has shown that measures have negatively affected the supply and demand chain of products and services across economic sectors [32,35,36], particularly affecting the food industry, hospitality, tourism, and aviation [32,36,37]. The fall in commodity prices has also caused a sharp drop in LATAM markets and currencies [38]. The International Monetary Fund estimated a 7% economic contraction in the region [23], expected to cause the worst recession in history [11]. In 2020 alone, 13.5% of people in LATAM became unemployed [39], and 22 million fell into poverty (an increase of 33.7% overall) [23], mainly affecting Argentina, Brazil, Mexico, and Peru [39].

Reducing the impact of the pandemic on the economy and people’s health and wellbeing requires effective policies that can help accelerate the return to normalcy. Vaccination has long been perceived as one of the main strategies to respond to the pandemic, triggering a massive effort to develop COVID-19 vaccines in record time. Governments of LATAM introduced COVID-19 vaccines in December 2020. With limited availability of resources and vaccine doses in many LATAM countries, tools that can help vaccination campaigns be carried out in the most effective and efficient way possible are extremely valuable. SARS-CoV-2 serology testing can provide important information to support decision-making regarding the containment and mitigation of COVID-19. According to information from other vaccine preventable diseases, within COVID-19 immunization policies, serology testing might play an important role in ensuring the effective planning, implementation and monitoring of immunization programs, as well as support endeavors to monitor and study the effectiveness of vaccines during immunization roll-out. This review will introduce the key aspects that shape the current pandemic scenario (the impact of virus mutations and the introduction of vaccination) while exploring potential avenues for the use of serology testing, based on current policy frameworks and available evidence.

This review has three goals: (1) to provide an overview of the role and value of serology testing as a tool to support COVID-19 immunization policies in LATAM, (2) to identify the barriers and challenges to the use and uptake of this tool in the current pandemic scenario, and (3) to provide an overview of the potential actions required to address these barriers and facilitate the uptake of this strategy in the region. International, national, and local health policy decision-makers involved in planning COVID-19 vaccination programs and strategies, the academic community, medical societies, as well as other stakeholders can use elements of this document to inspire further studies and build the necessary partnerships and alliances for collaborative actions.

## 3. Variants of Concern in Latin America

According to the WHO, as of October 2021, four variants of SARS-CoV-2 are classified as ‘variants of concern’ (VOC)—Alpha, Beta, Gamma, and Delta—first identified in the United Kingdom, South Africa, Brazil, and India, respectively [4]. According to current global genetic epidemiology, Delta has outcompeted other variants, including other VOCs in most counties. Nonetheless, subregional and country-level variations have been observed, particularly in LATAM, where the progression of Delta has been more gradual [4]. As of 26 October, Argentina, Brazil, Chile, and Mexico had identified cases of all four VOC; meanwhile, Colombia has only identified cases of VOC Alpha, Gamma, and Delta [4]. It is worth clarifying that this information refers to the identification of cases, but not necessarily the existence of community transmission of the VOC.

The emergence of virus mutations has been accompanied by concerns regarding their effect on transmissibility, severity of disease, immunity, and effectiveness of diagnostic methods. Evidence suggests an increased transmissibility of all VOC [40,41,42,43,44,45,46,47], and in particular a higher viral load in cases of Delta (when compared to Beta and non-VOC SARS-CoV-2 strain) [48,49] and a higher risk of pre-symptomatic transmission and secondary attack [48]. Regarding the impact on the severity of disease, studies have found a potential increased risk of hospitalization associated with all VOCs [50,51,52,53,54,55]. Notably, cases of infection from Gamma, Beta, and Delta were found to be at a considerably higher risk of hospitalization compared to cases from Alpha [56] and non-VOC SARS-CoV-2 strains [53,54]. Preliminary evidence points to the possible increased risk of hospital mortality associated with VOC Beta [57,58].

Evidence on the impact that VOC may have on immunity suggests a potential increased risk of reinfection for all VOC [45,51,52,53,54,57,58,59,60,61,62]. Researchers have reported a reduced neutralizing activity in VOC Beta [63,64,65,66] and Delta [67]. In the case of Gamma, evidence indicates only a moderate reduction in neutralizing activity [68,69]. Contrastingly, evidence points at retained neutralizing activity [70] and a similar risk of reinfection to the original virus strain in the case of Alpha [71,72].

Regarding the impact on vaccines effectiveness, it is worth noting that the evidence varies greatly depending on the VOC and the type of vaccine being studied. Having said that, evidence from a number of retrospective studies on periods of high incidence of the VOC and studies conducted in outbreak prompt settings indicates that vaccines grant similar protection against infection from Alpha [62,73,74] and Delta [62,74,75] to that expected for the vaccine. More importantly, evidence suggests the retention of the effectiveness of vaccines in reducing the severity of disease for Alpha [73,74,76,77], Beta [78,79,80,81], and Delta [82]. Nonetheless, other studies indicate a potential reduction in vaccine effectiveness against symptomatic disease for Beta [78,79,80,81] and Delta [82]. The impact of Gamma on vaccines effectiveness remains unclear.

WHO, PAHO, and the FDA have expressed concern regarding the potential loss of test performance as new variants emerge [83,84,85]. As a result, WHO recommends a diagnostic approach using different assays in parallel or multiplex assays targeting different viral genes [84]. PAHO recommends strengthening existing disease control activities and, where appropriate, adjusting public health and social measures to reduce the transmission of VOC [85].

## 4. Testing Strategies to Mitigate Impact: The Use of Serology Tests

There are currently two primary types of tests for COVID-19: (1) those used to diagnose acute infection through the direct detection of genetic material of the virus or specific viral antigens (molecular tests, antigen tests) and (2) those used to evaluate the antibody response (serology tests). Serology tests can provide essential information and evidence for research and policy-making purposes. Given the scope of this document, we will introduce the main characteristics of serology tests in this section.

Serology tests are designed to detect antibodies in the serum within days to weeks following acute infection [86,87]. The presence of antibodies can indicate that a person was infected with SARS-CoV-2, irrespective of whether the individual experienced severe, mild, or no symptoms. As such, serological data have an important place in the ongoing response to the COVID-19 pandemic, assisting surveillance activities, estimating epidemiological variables, assessing the effect of non-pharmaceutical interventions at the population level [88], and helping to evaluate vaccine efficacy and the immunological response triggered by both immunization and natural infection [89].

Serology tests vary depending on the choice of antibodies. Serology tests can measure three types of antibodies: immunoglobulin M (IgM), immunoglobulin G (IgG), and immunoglobulin A (IgA). However, the most commonly used are IgM and IgG, which are the two main isotypes of antibodies [13]. The specificities of tests measuring IgM/IgG and IgM are reportedly high, ranging from 96.6% to 99.7%, respectively [90,91]. According to a meta-analysis, sensitivity (for IgG and IgM tests) varies according to the testing method, but can be as high as 99%. Sensitivity was found to be higher (90–96%) for enzyme-linked immunosorbent assays (ELISA) and chemiluminescence enzyme immunoassays (CLIA), than for lateral flow immunoassays (LFIA) and fluorescence immunoassays (FIA), which range between 80% and 89% [91]. Evidence suggests that SARS-CoV-2 antibody production may differ from the typical scenario, with IgM and IgG antibodies tending to rise almost simultaneously [92,93,94]. In contrast to IgG, IgM and IgA antibodies have been found to decline more rapidly [94,95].

Serological tests also vary according to the viral antigens measured. Spike proteins (S) and nucleocapsid proteins (N) are the viral antigens used to detect antibodies for SARS-CoV-2. While testing positive for antibodies against either N, S, or receptor binding domains (RBD) can accurately indicate prior infection [96], recent evidence warns against the extensive use of N protein-based serology testing for determining potential protective immunity to COVID-19. Research has found that N protein-binding antibodies do not always correlate with the presence of S-RBD neutralizing antibodies [97].

In most cases, infection with SARS-CoV-2 initiates an adaptive antiviral humoral and cellular immune response, including B and T cell-mediated immunity [98,99,100]. The humoral response includes antibodies against specific viral antigens, such as N and S proteins. The S protein comprises two subunits, S1 and S2, the former containing the RBD that mediates the binding of the virus to cells. Evidence suggests that the RBD of S protein is the main target for neutralizing antibodies [99,101,102,103,104]. Tests targeting the S protein may provide higher sensitivity and specificity [102]. Serology studies might use the differential reactivity of S- and N-specific antibodies to help distinguish prior infection from vaccination, particularly for vaccines that produce antibodies only against the S protein [87]. Antibodies—including IgM, IgG, and IgA—against the S protein and its subunits can be detected starting at one to three weeks after infection [94,105], and until at least six months post-infection [106]. The sensitivity of these tests is higher from three weeks after symptom onset.

Notably, the neutralization assay, a lab-based test, is the gold standard for determining potential protective immunity, although a correlation with protection has not been established. This test can help (1) to increase understanding of immunity and potentially evaluate vaccine efficacy, (2) to determine the real number of infections by enhancing the serological diagnosis of asymptomatic infections, and (3) to identify eligible donors for a possibly beneficial convalescent plasma therapy [89]. Nevertheless, its broad implementation is limited because this test has a higher cost and requires a biosafety level 3 laboratory (a laboratory with permission to culture SARS-CoV-2-infected cells) [96].

An alternative that has received recent attention is the use of pseudovirus neutralization assays. Pseudovirus neutralization assays are a great alterative for highly pathogenic viruses such as SARS-CoV-2. A pseudovirus neutralization assay is a laboratory method used to study the effect of antibodies or drugs in preventing infection. This method uses a vector (pseudovirus) that has a similar conformational structure of the surface proteins and the ability to enter cells using the same mechanisms and receptors to that of the native virus. Pseudoviruses are, however, much safer to handle, since they cannot replicate, requiring only biosafety level 2 laboratories. Pseudovirus neutralization can be automated and standardized in laboratories across the world, as has been done for the human immunodeficiency virus [107].

This method has the potential for improving SARS-CoV-2 immunization policies, by providing an easy and accessible approach to classical serum neutralization assays, that can also easily be adapted to different SARS-CoV-2 variants and maintain a similar sensitivity and quantitative reliability [108]. Several important factors contribute to an effective vaccine response, including the presence of frequent virus mutations, the emergence of new virus strains, different vaccines in the market, and different patient characteristics (such as the use of different drugs and the presence of comorbidities). The use of neutralizing antibodies and indirect anti-RBD/anti-S assays can be useful to customize future approaches. It may also aid in choosing the ideal vaccine for a specific patient and the ideal period for a new shot. The evaluation of the cellular response against SARS-CoV-2 will soon also be available for clinical practice, which may also contribute to future decision-making [109].

Serological tests can be performed through laboratory-based assays and rapid diagnostic tests (RDT). While laboratory-based assays can generate more accurate results and provide qualitative and quantitative data, they have an increased turnaround time, higher cost, and require laboratory capacity [96]. Contrarily, RDTs usually require 15 to 30 min to process and can easily be implemented in decentralized settings. However, RDTs can only generate qualitative data and have shown wide variability in results. Qualitative data describe the absence or presence of antibodies in the sample, providing a simple answer on whether a person was once infected. Quantitative evidence, obtained through laboratory-based assays, provides more detailed information on the presence and level (or titer) of antibodies in the sample. The choice of tests is particularly important and should be carefully considered, taking into account the purpose of use, the cost, and the testing requirements and capacity in each context. Quantitative data are particularly important for studies aiming to understand the antibody response to natural infection and vaccination and to determine whether a person is eligible to donate convalescent plasma.

## 5. Introduction of SARS-CoV-2 Vaccines in Latin America

The pandemic has triggered a massive effort to develop COVID-19 vaccines in record time. Today, the general public perceives COVID-19 immunization as the most critical means of reducing the burden of disease, hospitalizations, and deaths, contributing to the return of normalcy and economic recovery. Nonetheless, immunization alone might not be sufficient to put a full stop to the pandemic and reach endemic status. Governments introduced COVID-19 vaccines in LATAM countries starting in December 2020. Unlike other regions of the world, countries in LATAM have proceeded independently regarding the procurement of doses and the principles that organize immunization rollout.

Launched in April 2020, COVID-19 Vaccines Global Access (COVAX) is a global platform to support the development, manufacturing, and distribution of COVID-19 vaccines [110,111], providing a procurement mechanism by which the platform purchases vaccines on behalf of countries [112]. By securing favorable purchasing deals, COVAX aims to contribute to addressing the unequal global distribution of doses. There are two types of countries participating in the COVAX acquisition program: (1) those in the position to self-finance and (2) those funded by the Gavi COVAX Advance Market Commitment program. While 14 countries in LATAM are self-financed [113,114], five middle- and lower-income countries are funded by COVAX [115].

Either through COVAX or independent agreements, countries in the region had contracted doses from 11 providers so far: AstraZeneca-Oxford, Janssen-Cilag, Moderna, Pfizer-BioNTech, Sputnik V, CanSino BIO, Sinopharm, Sinovac, Covishield, CureVac, and Novavax [110,116]. As multiple vaccines have been introduced in the region—diverse in type, manufacturer, and target population—studies evaluating the effectiveness and immune response to the various vaccines are particularly challenging yet necessary.

Given the limited supply of doses worldwide and the logistical challenge to immunize the whole population, countries have proceeded with immunization rollouts prioritizing different population groups. Most countries worldwide, including in LATAM, started vaccinating single groups, such as health care personnel and essential workers, and later proceeded to larger segments of the population based on different vulnerability parameters [117]. The criteria used for prioritization according to national COVID-19 immunization plans varies significantly between countries. As illustrated in Table 2, despite many international organizations advising countries to consider the risk of infection (indicated by a high prevalence of community transmission, for example) when prioritizing population groups, among the focus countries, only Argentina included this criterion in its national immunization plan [118]. In contrast, there is a broad agreement to prioritize based on age, comorbidities, and work/profession depending on higher exposure risk across Argentina [118], Brazil [119], Chile [120], Colombia [121,122], and Mexico [123].

Concerns regarding the safety of vaccines for service users have also accompanied immunization rollout in LATAM. While PAHO has recognized the need to strengthen national regulatory mechanisms to ensure that the introduced vaccines are safe and effective [124], the sense of urgency led many countries to administer vaccines that the WHO and/or national regulatory agencies had not approved for use yet [110]. The fear of contracting COVID-19 has led many people to resort to unauthorized distribution channels to access vaccines. Reports of counterfeit or unauthorized vaccines in circulation have emerged in countries such as Bolivia and Colombia [125]. Several news outlets have also reported on concerns from the general public regarding the motives behind decision-making, arguing that certain decisions on immunization policies may be political rather than evidence-driven.

Access disparities between and within countries are another source of concern. From a regional perspective, vaccination campaigns in LATAM have stalled due to unequal global access to vaccines and challenges surrounding vaccine production, distribution, and delivery [124,125,126], including considerable setbacks in the supply of doses acquired through COVAX [127]. Additionally, governments of many countries in the region must deal with the combination of a rugged terrain and an underdeveloped transport infrastructure, resulting in difficulties in appropriately delivering doses. While this has raised concerns regarding a possible vaccine divide between rural and urban communities [128], to this day, there is no clear evidence of this occurring. Furthermore, the decision to prioritize immunization in rural areas, regardless of local disease prevalence, has also been debated [129].

Delayed economic recovery is a particular source of apprehension, as evidence suggests that many LATAM countries will not reach full immunization of the eligible population until 2023 [128]. As of 28 October 2021, Chile is the leading country in the region, with 85% of its population immunized—76% fully vaccinated, and 8.3% partially. Among other focus countries, Brazil has managed to cover 74% of its population (55% fully vaccinated and 19% partially), followed closely by Argentina, with 73% immunized (56% fully vaccinated and 17% partially). Finally, Colombia has reached 58% coverage (40% fully vaccinated and 17% partially) and Mexico’s population is 55% immunized (42% fully vaccinated and 13% partially) [130].

## 6. Potential Areas of Use of Serology Testing and Seroepidemiological Evidence to Support Immunization Policies

Using serology tests and seroepidemiological data to support immunization policies and strategies across vaccine-preventable diseases is well-documented [131]. According to these studies, serology tests can provide evidence to support the planning, implementation, and monitoring of immunization policies and conduct post-marketing vaccine effectiveness studies to understand and evaluate the efficacy of the antibody response by different vaccines in the market (Table 3) [131,132,133,134].

In the context of post-marketing vaccine surveillance, serology tests have been used to (1) determine the duration of immunity after the primary series, (2) evaluate the need for and timing of booster doses, (3) evaluate dosing strategies, and (4) study the efficacy of vaccines across different population groups for vaccines with well-established correlates of protection [131,133,134].

Finally, stakeholders have also used seroepidemiological data to evaluate the effectiveness of immunization policies. Conducting this type of assessment requires evaluating the impact of immunization campaigns by monitoring population immunity over time. This activity is critical when there are recurrent outbreaks despite high immunization coverage. In the past, serology testing has helped in investigating possible causes of infection resurgence, such as those attributed to changes in vaccination schedule or formulation, and monitor progress towards elimination in due time [131,132].

Since the evidence gathered on the use of serosurveys and seroepidemiological data is limited to vaccine campaigns that target specific population groups, extrapolating these findings must be done carefully. In the current scenario, governments must administer COVID-19 vaccines to broader (if not all) segments of the population; thus, implementing serology testing as a policy within the pandemic should be assessed considering the conditions of each country and the cost–benefits of implementing this strategy in each context. Nonetheless, and as we will see in the next section, serology testing in the current pandemic scenario, could be used by the government to support the monitoring of infection and disease, including the middle- and long-term effects of COVID-19 on patients. Endeavoring to monitor population immunity over time can help researchers to investigate infection outbreaks and evaluate the progress of control measures, such as immunization. This information should be used to inform immunization policies, ensuring that resources are used in the most effective and efficient way. Targeted immunization activities and changes to immunization schedules can help to channel vaccine doses to where they are needed the most.

## 7. Current Guidelines and Recommendations on the Use of Serology Testing in the Region

From an international perspective, WHO, PAHO, FDA, and CDC have all provided statements and recommendations on the use of serology testing in the context of the COVID-19 pandemic. WHO has recommended against the use of serology testing for so-called “immunity passports” [135,136]. The use of serology testing has, however, been recognized for surveillance and research purposes [86]. According to WHO’s protocols for seroepidemiological studies, the use of serology testing can support five primary objectives: to (1) measure the seroprevalence of antibodies against COVID-19 in the general population to quantify the accumulated immunity, (2) estimate the proportion of pre-symptomatic, asymptomatic and subclinical infections in the population, (3) establish the risk factors for contracting the infection by comparing the exposures of infected and uninfected people, (4) accurately calculate the fatality rate, and (5) help to understand the kinetics of antibodies against COVID-19 better [137]. In alignment, PAHO recommends using serology testing for epidemiological investigations and seroprevalence studies, further calling for the use of tests measuring IgG and IgM antibodies [138].

Serology testing can also contribute to accurately forecasting the spread of COVID-19, providing essential evidence for optimal public policy measures. Different frameworks have been proposed for modeling the spread of COVID-19, including compartmental models, differential equation model, and branching point process models. The compartmental model Susceptible Exposed Infectious Removed (SEIR) is currently the most widely used model to forecast epidemic diseases such as COVID-19, despite the fact that it has been found to be less accurate than the Hawkes model (a branching point process model) [139]. Although many factors contribute to this discrepancy, the most significant factor seems to be that SEIR forecasts of future confirmed cases or deaths depend significantly on estimates of the total numbers of asymptomatic or mildly symptomatic cases [140,141], which is challenging to estimate. Most forecasting models require reliable estimates of the proportion of the population that remain susceptible to the disease and the proportion who have contracted the disease and may be, at least temporarily, immune. These particular estimates are difficult to calculate without population-wide testing [142,143]. Given that many COVID-19 cases are mild or showcase no symptoms, serology testing becomes a critical tool to provide accurate estimates, helping forecasting models to be parameterized accurately and obtain more reliable results.

In a statement, the WHO’s Director-General, Dr. Tedros Adhanom, recommended conducting seroprevalence studies to help understand the duration of immunity following both natural infection and vaccination and to evaluate the extension of the infection across different population groups [144]. The FDA and CDC are somewhat in agreement with this statement. On the one hand, these institutions recognize that serology tests can help to identify people who may have had a prior SARS-CoV-2 infection and have developed an immune response, aiding efforts to estimate the cumulative incidence of infection (or vaccination) in a community [87,145,146]. They also acknowledge that more research is needed to understand the robustness and durability of immunity, particularly following vaccination. However, the FDA and CDC state that the validity of using serology tests for this purpose is still pending [87], even though serology tests were used to evaluate vaccine efficacy during Phase III trials. Therefore, the FDA and CDC recommend against using antibody testing to assess a person’s immunity or protection from COVID-19 following vaccination and the need for vaccination in unvaccinated individuals [87,146].

At the regional level, the Argentinian Consensus on the Use of Diagnostic Tests for SARS-CoV-2 [147] recommends implementing seroprevalence studies to assess the evolution of the pandemic and using serology testing to identify possible candidates for donating convalescent plasma for convalescent plasma therapy. The FDA shares this position [145]. Moreover, this consensus stands out by recommending the use of serology testing to conduct retrospective diagnoses of asymptomatic infections or infections that were not detected earlier to identify the association of infection and late complications [147].

When analyzing the countries of focus, it is worth noting that none have included serology testing in their national immunization plans. However, in Argentina [148], Brazil [149], Chile [150], and Mexico [151], serology testing has been recognized and implemented by different research groups to estimate the prevalence of infection, study the immune response to the disease, and identify individuals that might be protected after natural infection or vaccination, among other purposes. As of 20 October 2021, according to the SeroTracker, Brazil, Chile and Mexico have conducted country-/territory-wide serosurveys as well as local studies. In contrast, Argentina has only conducted local serosurveys [152]. Notably, a study on vaccines effectiveness in nearly 60,000 individuals in Chile found that IgG positivity for CoronaVac recipients was considerably lower after the first and the second doses (28% and 77%, respectively) than for the Pfizer–BioNTech’s mRNA BNT162b2 vaccine recipients, which record a seropositivity of 80% after the first dose and 95% after the second dose. This study supports the argument that monitoring seropositivity over time can provide data to reassess future vaccination rollout strategies [153].

Finally, while Argentina [154] and Colombia [155] have followed the recommendation provided by international guidelines against the use of serology testing to diagnose acute infection [87,138,145,146,156], Brazil recognizes the use of serology tests as an auxiliary diagnosis tool [149].

## 8. Challenges and Barriers to the Use of Serology Testing to Support Immunization Policies in Latin America and the World

Several international organizations and the academic community have voiced their concerns regarding the challenges and barriers to the use of serology testing in the context of immunization policies and the broader response to the COVID-19 pandemic. These challenges can be categorized into two main groups. The first group includes the intrinsic limitations of serology testing. The second group points to the challenges surrounding the use of serology testing in the context of immunization policies, surveillance and monitoring activities, and epidemiology studies.

### 8.1. Challenges Related to the Limitations of Serology Testing

Some researchers observed considerable variations in the results of SARS-CoV-2 seroprevalence studies. Studies may underestimate the prevalence of SARS-CoV-2 for several reasons. The first and perhaps more apparent reason results from the choice of assay [157]. Not only do the sensitivity and specificity of serology tests vary between manufacturers [158], but variations in test performance can be observed depending on the choice of antibodies and antigens measured [157], the type of assay and viral protein measured, the type of sample (blood or plasma) used, and the sample collection process [149]. Another issue is the use of quantitative versus qualitative tests. The former can provide more space to detect low-level antibodies, which the latter may not. Moreover, the difficulty in detecting mild or asymptomatic cases, resulting in missed community cases and several demographic factors such as age, sex, and ethnicity, may influence test calibration [157].

Cross-reactivity has been a source of concern regarding the use of certain serology tests in prevalence studies [149]. While emerging evidence suggests that cross-reactivity is low to other coronaviruses and influenza A and B [159,160,161,162], further studies are needed to determine whether these results will persist through longitudinally collected serum samples and to confirm that emerging SARS-CoV-2 variants, especially those affecting the S protein, will not result in changes in cross-reactivity [160,163].

Finally, while the use of serologic testing to identify, evaluate and understand the immune response from natural infection is well accepted, many argue that serology tests are not entirely validated to assess the level of protection provided by COVID-19 vaccination [87,146]. Moreover, since only a few serology tests can distinguish between the antibody response triggered from natural infection and vaccination [147], the use of serology testing to evaluate COVID-19 vaccine-induced immunity remains a contested idea.

### 8.2. Challenges Related to the Use of Serology Testing to Support Immunization Policies and Estimate Epidemiological Variables

Using serology testing to support immunization policies is primarily limited by gaps in the knowledge and understanding of (1) the immune response triggered by infection and vaccination and (2) the subsequently conferred immunity. How long antibodies against SARS-CoV-2 persist in the body, the titers of protection and the conditions that lead to protection remain unknown. While some studies indicate that IgG antibodies, including IgG against the S and N proteins, persist for at least nine to ten months after infection in most cases [106], other studies have reported an absence of IgG antibodies following infection in approximately 5–10% of cases [164,165]. Moreover, since antibody persistence has been noted to vary between assays, our understanding of the antibody response may be limited by the choice of tests [166]. Notably, the conditions that lead to protection and reinfection, including the roles of humoral and cellular immune responses, also remain unclear. Nevertheless, recent evidence indicates that previous infection leads to a substantially reduced risk of reinfection in the following six to seven months after infection [167,168,169,170].

Determining if infection or vaccination confers an antibody response that grants immunity essentially depends on whether correlates of protection are available. A correlate of protection is an immunological measurement (an immune marker) used to predict protection against disease or infection reliably [171,172]. Determining correlates of protection may be challenged by recorded differences in the antibody response found across immune-competent populations. Studies have documented, for example, that patients who had experienced more severe symptoms had also developed a more robust antibody response, exhibiting higher titers and longer persistence of IgM, IgG, and IgA antibodies [173,174]. Studies have also reported discrepancies in reinfection rates related to age group. While a study conducted on young males found that previous infection reduces the incidence rate of reinfection by 82% [175], another documented a reduction of only 47.1% among adults aged 65 years and older [168]. Sound correlates of protection will have to account for differences of this nature.

Nonetheless, in recent months, significant evidence has been developed on correlates of protection. Although there are documented cases of reinfection [176,177], several studies indicate that individuals who have SARS-CoV-2 antibodies are less likely to experience reinfection than individuals who do not have such antibodies [167,170,178,179,180,181,182]. Some studies suggest that previous infection may reduce the risk of reinfection by 80% to 84% [167,168], and that lower SARS-CoV-2 IgG titers (or their absence) and lower levels of neutralizing antibodies may correlate with a higher risk of reinfection [175]. Notably, a recent study using data from 171 cases of SARS-CoV-2 infection and 1404 non-cases demonstrated that higher anti-spike IgG, anti-RBD IgG, and neutralizing antibody titers are all associated with a lower risk of symptomatic disease. However, the same study also found that none of the serological measurements showed a correlation with protection against asymptomatic infection or symptomatic illness with mild upper respiratory symptoms [183], further confirming the observation that infection remains possible in fully vaccinated individuals, despite high effectiveness against more severe forms of the disease (such as those causing hospitalization or death) as reported by COVID-19 vaccine clinical trials and real-world evidence from vaccine rollout programs [76,184,185,186,187,188,189,190,191,192,193,194]. According to the results of this study, there is no single threshold value indicative of protective immunity. Instead, there is a decrease and increase in the probability of infection relative to a higher and lower immune response, respectively [183]. Feng et al. (2021) instead provided antibody estimates that correspond with 50% to 90% vaccine efficacies.

While evidence of this nature supports the argument that post-immunization antibody levels might be used as the basis for a correlate of protection, the necessary next steps toward achieving consensus on this measurement include: (1) establishing comparable antibody measurements across laboratories, (2) agreeing on a neutralization assay to serve as the gold standard, (3) calculating, where possible, the protective threshold in Phase III studies, (4) convening stakeholders to reach a consensus despite discrepancies between studies, and (5) verifying that the correlate of protection will apply to new variants using appropriately adapted assays [195].

Given the discrepancies in the antibody response across populations, the absence of consensus on clinical markers of correlation, and the lack of knowledge of the impact of emerging variants on immunity, additional studies are required to provide sound correlates of protection. There is a strong need for standardized processes and more extensive longitudinal and multicenter studies that include different population groups. Efforts to address knowledge gaps should emphasize the adequacy of the study design and its standardized implementation to ensure the comparability of data across countries. While the WHO has taken the first steps to standardize the different assays available in the market by suggesting the use of a single measurement unit (BAU/mL), urging different in vitro diagnostic companies to standardize their assays [196], there is also an imperative need to translate current evidence into recommendations and guidelines addressing the strengths and weaknesses of tests in this evolving scenario. Considering the financial constraints faced by many LATAM countries, stakeholders from the private and public sectors should invest to ensure the availability of adequate human and technological capacity to implement these studies. As new evidence becomes available, international organizations, professional societies and the academic community should help to translate this evidence into sound recommendations to ensure decisions on the use of serology testing are based on the best available evidence, across the different stages of immunization. The absence of guidelines poses a risk to the safety of service users and increases concerns about inequalities. In the absence of guidelines, only those in a position of financial and expertise privilege will be able to effectively use serology tests.

Once correlates of protection are available, in partnership with research institutes, governments could conduct serosurveys on vaccinated individuals from specific population groups and the general public to determine the duration of immunity and the need and timing of booster doses. In addition, serosurveys could help us to assess the effectiveness of different vaccine formulations as they become available and are being introduced in different epidemiological scenarios, including those with new virus variants. The implementation of serosurveys to evaluate and monitor vaccine effectiveness should be considered as a complementary tool to infection studies and carefully considering the prevalence of infection in the community when interpreting data.

Besides the presented knowledge gaps, implementing serosurveys also faces challenges related to time, technology, logistical, and financial constraints [131]. These challenges may limit the use of serology testing to targeted activities rather than broad policies. Serosurvey implementation requires (1) integration between clinical, laboratory, and epidemiology aspects, (2) appropriate study design and optimal sample size, (3) adequate laboratory capacity, (4) appropriate laboratory methods, and (5) standardized and validated procedures [132,147,197]. Considering the financial constraints faced by many LATAM countries, public–private partnerships may become an essential piece of the puzzle, as these would allow higher investment in capacity and resources to implement these studies. It is worth noting that the choice of serology test is key to ensuring the validity of results, comparability, and integration of data across studies and countries. Evidence points to serology tests measuring IgG antibodies against S-RBD as the best available to inform SARS-CoV-2 vaccine-associated immunity [198,199,200], yet many commercially available kits detect binding antibodies against the N protein. While these tests may not be the best alternative to evaluate vaccine-induced immunity, they may still be used to detect prior exposure to the virus [97]. Manufacturers must ensure that tests’ properties are clearly disclaimed, so that service users and researchers can select a test according to the purpose of use.

Finally, as multiple vaccines, diverse in both manufacturer and type, are currently being introduced to individuals who may or may not have had prior infection, studying and evaluating antibody production and vaccine-induced immunity becomes exceptionally complicated. Research institutes and the academic community must pay particular attention to studies that can help to identify and understand potential differences in the kinetics of the immune response and antibody dynamics against SARS-CoV-2 and vaccination.

## 9. Conclusions

Evidence and current national and international guidelines and recommendations highlight the potential benefits of using serology testing as a strategy to support COVID-19 immunization policies and the broader policy response to the pandemic. Serology tests have successfully helped to develop evidence and provide critical information for decision-making in the past. While considerable financial and logistical constraints characterize the current scenario, the benefits of using serology testing might outweigh the medium- to long-term costs.

Serology tests are a valuable tool to help us understand the antibody response triggered by natural infection and vaccines, as well as to provide the necessary evidence to design better and more effective immunization policies. With a concerning shortage of vaccines in many LATAM countries and the widely accepted notion that immunization is vital for the control of the pandemic and the return to normalcy, information that can contribute to implementing effective immunization strategies is particularly critical to safeguard the economic recovery of countries and prevent soaring inequalities in the region.

Nevertheless, the full use of serology tests in this context requires developing the necessary evidence to address critical knowledge gaps. For this purpose, the commitment and collaboration of both private and public stakeholders and the academic and international communities are of utmost importance.

## Figures and Tables

**Table 1:** Impact of the pandemic in focus countries: Argentina, Brazil, Chile, Colombia, and Mexico.

Country	Deaths Reported by 100 K Habitants [6] (as of 27 October 2021)	14-Day Notification Death Rate per 1 M Inhabitants [10] (as of 28 October 2021)	Case Fatality Ratio * [7] (as of 28 October 2021)
Argentina	258	7.96	2.2%
Brazil	287	21.79	2.8%
Chile	199	5.96	2.2%
Colombia	253	8.72	2.5%
Mexico	225	24.19	7.6%

* Case-fatality ratio: number of deaths per 100 confirmed cases. Source: based on available data from the Inter-American Development Bank, the European Center of Disease Prevention and Control, the World Health Organization, and the Johns Hopkins University and Medicine Coronavirus Resource Center.

**Table 2:** Prioritization criteria according to national COVID-19 immunization plans in focus countries.

Prioritization Criteria	Argentina	Brazil	Chile	Colombia	Mexico
People at high risk of infection *	✓				
Age (60–70+)	✓	✓	✓	✓	✓
People at risk of severe disease **	✓	✓	✓	✓	✓
People in vulnerable conditions	✓	✓		✓	
Health care workers	✓	✓	✓	✓	✓
Essential workers	✓	✓	✓	✓	

* People at a higher risk of contracting the disease due to high transmission prevalence in their community. ** People that have a higher risk of developing severe symptoms due to comorbidities or debilitating factors. Source: based on reviewed resources [118,119,120,121,122,123].

**Table 3:** Potential areas where using serology testing can support immunization activities.

	Areas/Activities for the Possible Use of Serology Testing	Examples of Use across Vaccine-Preventable Diseases
Post-marketing surveillance of the efficacy and duration of protection of vaccines	Determine the duration of immunity after the primary series.	Diphtheria Hib Meningococcus Pertussis Tetanus
	Determine the need for and timing of booster doses and evaluate doses strategy.	
	Determine the efficacy of a vaccine across populations.	Pneumonia HPV
Immunization policy planning	Estimate burden of disease.	Hepatitis B Rubella Hepatitis A Measles Varicella Yellow fever
	Estimate theoretical herd immunity thresholds derived from both immunization and natural infection.	Hepatitis B Measles Rubella Poliomyelitis
	Guide decision-making regarding the need for supplemental immunization activities and changes to immunization schedules.	Measles Rubella Poliomyelitis
	Identify prioritization groups for the first stage of vaccination rollout.	
Monitoring effectiveness of immunization policies and outbreak vigilance	Monitor population immunity over time, especially useful in the absence of virus circulation.	Hepatitis B Measles Rubella Poliomyelitis Tetanus
	Monitor progress towards disease elimination.	
	Identify groups with gaps in immunity and with active transmission to target immunization campaigns.	
	Investigate causes of disease resurgence.	Diphtheria Hib Meningococcus Pertussis
	Assess the risk of outbreaks and identify high-risk population subgroups, especially useful in the absence of virus circulation.	
	Evaluate the impact of campaigns and effectiveness of immunization programs, particularly when there are continued outbreaks despite high reported coverage.	Measles Rubella Poliomyelitis
	Estimate vaccine coverage, only when there is absence of virus circulation and reliable record-keeping.	Tetanus (potentially) Hepatitis B (potentially)

Source: based on reviewed resources [131,132,133,134].

## Data Availability

Not applicable.
